# Effects of adaptation and synaptic plasticity on synchronization of coupled oscillating neurons

**DOI:** 10.1186/1471-2202-12-S1-P240

**Published:** 2011-07-18

**Authors:** Josef Ladenbauer, LieJune Shiau, Klaus Obermayer

**Affiliations:** 1 Department of Software Engineering and Theoretical Computer Science, Technische Universität Berlin, 10623 Berlin, Germany; 2 Department of Mathematics, University of Houston, Houston, TX 77058, USA

## 

Synchronized oscillating activity in cortical circuits is a prevalent phenomenon and considered to be fundamental to cognitive function, selective attention and consciousness. In the present study we describe how spike-frequency adaptation (SFA) affects the synchronization properties of coupled neurons driven to repetitive firing, in dependence of axonal conduction delays and spike-timing-dependent plasticity (STDP).

We analyze networks of adaptive exponential integrate-and-fire (aEIF) neurons by applying phase reduction theory based on the assumption of weak coupling [1]. We calculate infinitesimal phase response curves (PRCs) and combine these with conductance based AMPA- and GABA-like synapses, in order to obtain interaction functions which determine the dynamics of the reduced phase network. This allows for the identification of stable synchronous and out-of-phase locked states of coupled pairs. The aEIF model involves a subthreshold as well as a spike-triggered adaptation parameter which can be related to the M-current and the after hyperpolarization current, respectively, both producing SFA. The former causes a transition of the PRC from type I (only advancing) to type II (advancing and delaying) whereas the latter determines its skewness.

**Figure 1:**
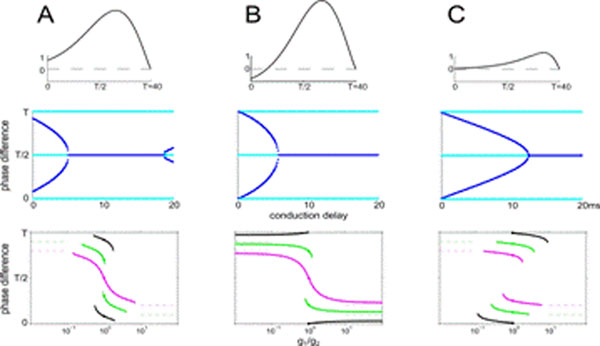
Top: Infinitesimal PRCs with low SFA (A), large subthreshold adaptation (B), large spike-triggered adaptation component (C). Middle: Stable (dark blue) and unstable (light blue) phase locked states of excitatory pairs in dependence of the conduction delay. Bottom: Stable phase locking of excitatory pairs with 0, 4, 8 ms conduction delay (black, green, magenta) in dependence of the coupling strength ratio.

We find that SFA in coupled excitatory pairs synchronizes oscillations as long as conduction delays are negligible, in agreement with [2]. When the delays are increased, the stable states shift from in-phase towards anti-phase locking (see Figure 1, middle). Inhibitory pairs show stable synchrony independent of the conduction delay, as well as anti-phase locking for short delays. Our analysis reveals that excitatory-inhibitory pairs do not phase lock if both neurons have type I PRCs. Type II pairs on the other hand have stable phase locked states even for diverging synaptic strengths (see Figure 1, bottom). When the coupling strengths are modified under the influence of a symmetric STDP rule, such pairs phase lock at a phase difference slightly above the conduction delay, for small delays. We complement these results on pairs by numerical simulations of phase as well as spiking aEIF networks, respectively, demonstrating that STDP promotes pairwise phase locking (clustering) in networks of excitatory neurons with low spike-triggered adaptation, where the number of clusters depends on the conduction delay.
